# Infants Infer Social Relationships Between Individuals Who Engage in Imitative Social Interactions

**DOI:** 10.1162/opmi_a_00124

**Published:** 2024-03-05

**Authors:** Vanessa Kudrnova, Elizabeth S. Spelke, Ashley J. Thomas

**Affiliations:** University of College London; Psychology, Harvard University

**Keywords:** imitation, infant cognition, social development, social relationships

## Abstract

Infants are born into rich social networks and are faced with the challenge of learning about them. When infants observe social interactions, they make predictions about future behavior, but it is not clear whether these predictions are based on social dispositions, social relationships, or both. The current studies (N = 188, N = 90 males) address this question in 12-month-old infants and 16- to 18-month-old toddlers who observe social interactions involving imitation. In Studies 1 and 3, infants and toddlers expected that imitators, compared to non-imitators, would respond to their social partners’ distress. Likewise, they expected the targets of imitation, compared to non-targets, to respond to their partner’s distress. In Study 2, these expectations did not generalize to interactions with a new partner, providing evidence that infants learned about the relationships between individuals as opposed to their dispositions. In Study 3, infants did not make predictions about responses to laughter, suggesting that infants see imitation as indicative of a specific kind of social relationship. Together, these results provide evidence that imitative interactions support infants’ and toddlers’ learning about the social relationships connecting unknown individuals.

## INTRODUCTION

Human infants face the challenge of learning about the people and relationships that make up the large social networks into which they are born (Fiske, 1992; Kaufmann & Clément, 2014; Tatone, 2017; Thomas, n.d.; Thomsen & Carey, 2013). Over the first year of life, infants’ social skills expand (Tomasello, 2016), as does the number of people with whom they interact (Helfrecht et al., 2020; Hrdy & Burkart, 2022). As prolific observational learners with limited language comprehension (Spelke, 2022), infants must therefore infer dispositions and relationships of the people around them by observing their social interactions.

One challenge when observing social interactions is that the same behavior can be indicative of either a person’s social dispositions or of their relationships. Different generalizations follow from the two interpretations. For example, imagine you see an adult playfully imitating a child. If you explain the adult’s behavior by inferring a disposition (e.g., the adult is ‘playful’ or ‘kind’) then predictions about the adult’s behavior toward other children are warranted, and predictions about the child’s behavior toward the adult are not. In contrast, if you explain the adult’s behavior by inferring a relationship between the adult and the child, predictions about the child’s and the adult’s behavior toward each other are warranted, but predictions about the adult’s behavior toward other children are not. The present studies investigate whether 12-month-old infants and 16- to 18-month-old toddlers make inferences about prosocial dispositions or affiliative relationships, or both, when they observe social interactions that involve imitation. If observed acts of imitation are interpreted by infants as indicative of social relationships, we reasoned that infants’ predictions about future social behaviors should be (1) symmetrical: infants should have expectations about the future behavior of both those who imitate and those who are imitated; (2) constrained to the individuals involved in the interaction: infants should have no expectations about the future behavior of either party toward other individuals; and (3) constrained to social behaviors that are more likely to occur in established social relationships: infants should have no expectations, for example, concerning the object-directed actions of either individual. In contrast, if infants infer a prosocial disposition from imitation, we reasoned that their predictions about future behavior should (1) apply to imitators but not those who were imitated, (2) extend to situations in which the imitator interacts with new individuals, and (3) extend to other social situations.

Of course, the way that people act in a single social interaction can be diagnostic of both the person’s dispositions and their relationship to others. However, some social behaviors, including laughing, speaking and smiling, are often directed to strangers and may thus be more indicative of dispositions (Devereux & Ginsburg, 2001; Owren & Bachorowski, 2003). Many other social behaviors, such as close bodily contact, comforting, synchronizing, or displays of distress, may be more common in relationships and thus be more indicative of and expected in relationships (Buchheim et al., 2009; Davidov & Grusec, 2006; Fiske, 1992; Seyfarth & Cheney, 2003; Silk, 2007; Sorokowska et al., 2021). Infants’ predictions may differ depending on the social actions they observe: when they infer dispositions, they should more readily make predictions about reactions to behaviors that occur between strangers (e.g., laughter). However, when they infer social relationships, they should more readily make predictions about actions characteristic of such relationships (e.g., displays of, and responses to, distress). Accordingly, we reasoned if infants infer prosocial dispositions from imitation, their generalizations should extend to many contexts (e.g., responses both to distress and to laughter, both with new people as well as the original partners). If infants make inferences about relationships when they observe imitation, their predictions may be constrained to interactions that occur more often in relationships (i.e., responses by the imitated party to the distress of their imitator).

In past research, infants inferred relationships after they observed responses to distress and after seeing actions that implied saliva-sharing, both of which are cross-cultural and cross-species cues of relationships (Cheney & Seyfarth, 1992; Fiske, 1992). In one study, 15- to 17-month-old toddlers used responses to distress to infer triadic closure: they expected that two small characters who had been comforted by the same large character, or two large characters who had comforted the same small character, would affiliate by approaching one another and coordinating their actions; (Billingsley et al., 2019). In another study, infants as young as 8 months predicted that individuals who shared saliva would be more likely to respond to one another’s distress, compared to individuals who shared a toy or who touched one another in a non-affiliative way (Thomas, Woo, et al., 2022). Importantly, infants’ predictions were constrained to the participants in the interactions: when an individual who had not been part of the interactions expressed distress, infants did not expect the saliva-sharer to respond. Infants’ expectations were also constrained to social actions that typically occur between socially related individuals: they did not expect saliva sharers, as opposed to other social partners, to respond to their partner’s speech or to a request for a ball: a response that may occur toward strangers. In another study, 4- and 12-month-olds expected a woman to comfort a crying baby as opposed to approaching a pile of laundry (Jin et al., 2018). They did not expect the woman to approach the baby when it was laughing. While the relationship between the woman and baby was not shown directly, other cues, including the size of the woman and the sound of a baby crying, may have prompted babies to infer a caregiving relationship. Their expectations of response were constrained to crying. Together, these findings suggest that infants infer social relationships rather than individual dispositions after viewing specific social actions that commonly occur between individuals in established relationships.

On the other hand, infants seem to infer dispositions after seeing interactions that often happen between strangers or in established relationships. For example, infants reach for and look longer at helpers: prosocial individuals who adopt the instrumental goals of others (see Woo et al., 2022 for review). They also look longer at characters who approach other characters compared to those who approach and ‘push’ other characters (Geraci et al., 2022). They reach for or look longer at those who distribute resources equally or fairly (Geraci, 2022; Geraci & Surian, 2011, 2023), those who defer in conflicts by letting someone else pass (Thomas & Sarnecka, 2019) and protectors over aggressors (Kanakogi et al., 2013, 2017). These preferences have been interpreted as evidence for ‘an innate moral core’ (Hamlin, 2013; Woo et al., 2022) in which infants evaluate others based on whether they are morally good or bad. These preferences have also been used to argue that infants prefer to interact with those who adopt the goals of others (Powell, 2022) or that infants are more interested in those who act to produce new outcomes (Spelke, 2022). The implication behind all of these interpretations is that when infants see someone being prosocial, they are motivated to interact with them because they infer a disposition: someone who acts pro-socially toward one person is likely to act pro-socially toward others, including the infant herself. Interestingly, in one study, 12-month-old infants did not reach more often for those who responded to distress compared to those who failed to do so, possibly because responses to distress are more indicative of social relationships than of dispositions (Thomas et al., 2020).

What category does imitation fall into? While infants show both preferential looking and reaching for imitators (Powell & Spelke, 2018b), it is unclear whether infants see imitation as a cue to dispositions or relationships. In one study, eight-month-old infants were shown two social groups (3 characters coordinated their actions by moving in a synchronized circle). During test, they expected members of the groups to imitate one another as opposed to members of the other group (Powell & Spelke, 2013). These expectations could not be the result of inferred dispositions, because when the groups were established, members of both groups performed the same actions. The key variable in this study is whether characters imitated other characters who they had previously coordinated with, relative to characters with whom they had not coordinated. Infants also expect that members of groups, cued by common labels and synchronization, will preferentially support one another (Cirelli et al., 2014; Jin & Baillargeon, 2017; Ting et al., 2019).

Moreover, 4-month-old infants expect imitators to approach the target of their imitation, consistent with inferences of social relationships (Powell & Spelke, 2018a). However, in line with dispositional inferences, their expectations are asymmetric: They do not expect the targets of imitation to approach the individual who initiated the imitation. Likewise 4-month-old infants preferentially look at imitators, and 12 month-old infants reach for imitators, and neither age group has demonstrated any preference for the targets of imitation (Powell & Spelke, 2018a; Thomas et al., 2020; Thomas, Saxe, et al., 2022). Infants’ distinction between initiators and responders in prosocial interactions could be evidence that younger infants attribute dispositions to imitators. That is, being the individual who initiates imitation may be evidence of an intent to be prosocial, whereas being the target of imitation can happen unintentionally. On the other hand, the most relevant relationships to infants may be caregiving relationships, which are often asymmetrical in obligations and interactions (Powell, 2022). Thus, young infants may see imitation as indicative of social relationships, but they may view imitative interactions as asymmetrical until they are able to become more active social partners themselves.

One set of results strongly suggests that 12-month-old infants see imitation as a cue to relationships: They distinguish between the targets of their parent’s imitation and the targets of a stranger’s imitation (Thomas, Saxe, et al., 2022), reaching for and expecting social engagement from the targets of their parent’s imitation but not from the targets of an unfamiliar adult’s imitation. Importantly, in this study, the parents had a friendly and contingent interaction with two puppets, but they only imitated one puppet. These results suggest that the infants see their parent’s imitation as more relevant to themselves than the imitation of strangers, potentially because they see imitation as a distinct cue of relationships: a cue that thereby differs from social cues to friendliness or social speech, which frequently is directed to strangers.

This body of work, along with others, suggests that infants make inferences from observed social interactions. There is some evidence that infants see some social behaviors as indicative of relationships, while other social behaviors are indicative of dispositions. However, the experiments do not reveal how infants’ view acts of social imitation. On the one hand, the preferences for imitators suggest that they attribute a prosocial disposition to imitators. If so, then infants should expect that future prosocial behavior will be applied to other individuals. On the other hand, 12 month old infants infer that they themselves are related to new individuals who have been imitated by their own parents, suggesting that they see imitation as a cue to social relationships. If so, then infants’ predictions should be constrained to the individuals they observe imitating one another. Moreover, their predictions about future actions should be constrained to actions that occur in social relationships and should exclude actions that commonly occur between strangers. Previous studies have not systematically tested these possibilities.

A series of three experiments investigated whether infants’ inferences about imitation were based on inferred relationships or inferred dispositions. In these studies, we show infants two sets of interactions between a central human actor and two flanking puppets. In the actor condition, one puppet imitates the central person, and the other puppet does not, allowing us to compare infants’ predictions about imitators and non-imitators. We used vocal imitation, with no obvious instrumental goal, because previous studies have shown that infants view vocal imitation as social, increasing the likelihood that infants will view the present interactions as socially motivated. In the target condition, one puppet is imitated by the central person and the other is not, and then the central person expresses distress. These events allow us to compare infants’ predictions about those who are imitated compared to those who are not imitated, by determining who infants looked to first: a measure of their expectations about who would respond (see Thomas, Saxe, et al., 2022).

In Study 1, we ask whether 11–12-month-old infants and 16-18 month old toddlers expect imitators and their targets to respond to their social partner’s distress. We chose to test infants and toddlers in our first study because previous studies tested in these age ranges (Spokes & Spelke, 2017; Thomas et al., 2020; Thomas, Saxe, et al., 2022; Thomas, Woo, et al., 2022) (Spokes & Spelke, 2017; Thomas, Saxe, et al., 2022). In Study 2, we ask whether infants’ expectations are limited to the individuals involved in the original interactions, or whether they expect imitators to be responsive to anyone who expresses distress. In Study 3, we ask whether infants’ expectations are limited to responses to distress—i.e., social reactions that occur more often in relationships—or whether their expectations extend to responses to laughter: a social behavior that occurs commonly in interactions with strangers. We reasoned that if infants failed to generalize beyond the individuals in the interaction and beyond comforting, then they more likely inferred a social relationship between the parties in the imitative interaction.

## STUDY 1

Pre-registered here: https://osf.io/e2dzw/?view_only=dca950dd78214fd4b215b8afc59af62b.

### Methods

#### Participants.

Thirty-four infants (M_months_ = 11.94, SD = .26, range = 11.6–12.4, 10 female and 24 male) and 31 toddlers (M_months_ = 17.52, SD = .67, range = 16.60–18.67, 20 female and 11 male) participated in the study. One infant’s data was excluded in the target condition because of video quality, and two infants’s data was excluded in the actor condition, one because of video quality and one because of inattentiveness. One toddler’s data was excluded from the actor condition because of inattentiveness.

#### Materials and Procedure.

**Figure 1.**
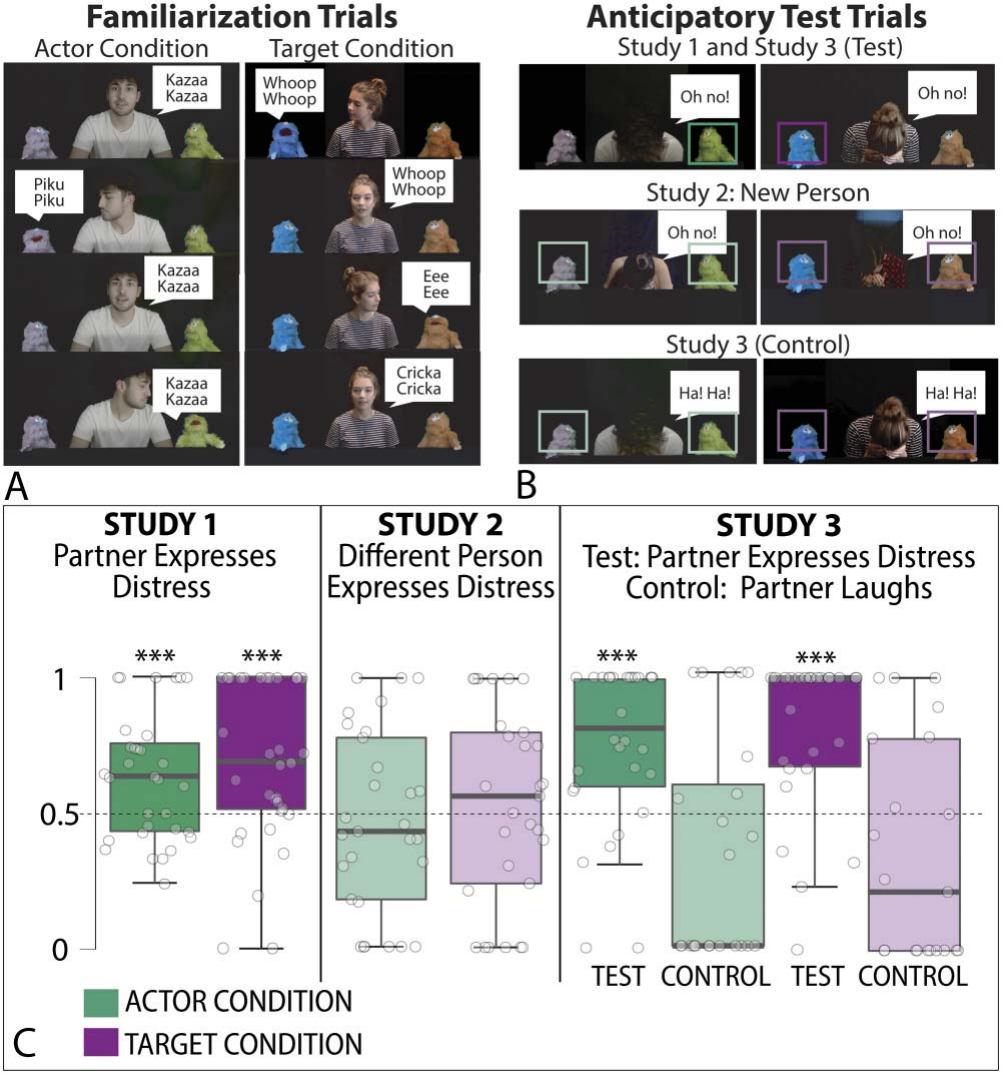
(A) Familiarization used in the three experiments. (B) Anticipatory Test Trials. Boxes added around puppets to show predicted looking patterns. (C) Boxplots of the proportion of time infants spent looking at puppets in imitative interaction during the anticipatory looking trials across three experiments. White dots represent the proportion of time that individual infants’ spent looking at the puppet who imitated compared to the puppet who did not imitate; or toward the puppet who had been imitated compared to the puppet who had not been imitated. Black bars are medians. Created using JASP. *** indicate Bayes Factor >10.

All stimuli can be found on the Open Science Framework (https://osf.io/e2dzw/?view_only=dca950dd78214fd4b215b8afc59af62b). For all the familiarization events and test events, we made films using different colored ‘monster’ puppets that were 14” tall. Each infant saw two conditions: In the Actor Imitation Condition, infants saw a scene with an adult person flanked by two different colored puppets. First, the person made a noise (e.g. ‘kazaa, kazaa’). Then, after the person turned to one of the puppets, the puppet made either the same or different noise (e.g., ‘piku, piku’, spoken by a different voice). Then, the person looked forward and made the same noise that it made in the beginning of the scene. Finally, the person turned toward the other puppet who either made the same noise (e.g., ‘kazaa, kazaa’, spoken by a different voice) or a different noise, such that one puppet imitated the person, and one puppet did not (See Figure 1). In the Target Imitation Condition, the roles of the person and the puppets reversed. Infants saw a different person flanked by a different pair of puppets. First, one puppet vocalized, and the person responded by making the same or a different sound. Then, the other puppet vocalized, and the person responded in the opposite manner. Thus, one puppet was imitated by the central person and one puppet was not. In both conditions, the central person had a friendly vocal interaction with both puppets but had an imitative interaction with only one puppet (See Figure 1).

After each familiarization condition, participants were presented with three test trials. In the main confirmatory Anticipatory test trial, the actor expressed distress (the actor frowned, said, “Oh no!” and put their head in their hands, see Thomas et al., 2022). The dependent variable was anticipatory looking: after the expression of distress, the scene was paused for 8 seconds during which time infants’ anticipatory looks and duration of gaze toward the left and right puppet were coded. Next came a social preference test (8s), in which the puppets appeared on-screen and only one voice called out to the baby, saying, “Hi baby, hi!”. Finally, infants saw a general preference test (8s) in which the puppets jiggled on the screen with upbeat music in the background. The order of conditions, gender of actors, puppet identity (e.g., blue puppet imitator vs. orange puppet is imitator), and side of imitator/imitated puppet was counterbalanced across participants. All infants saw both the actor and target conditions (See Figure 1).

Experimenters met infants and their parents via video chat. Parents were instructed to have their infants sitting in a highchair or on their lap and to remain neutral during the experiment. The videos were presented to the infants on their parent’s screens, and their faces were recorded using the video chat software.

#### Coding, Analysis and Stopping Rule.

Coding was done offline with coders unaware of the identity of the imitator/imitated puppets as well as the order of conditions. Coders recorded the direction of babies’ first looks immediately following the central character’s expression of distress. Second, coders recorded looking time to the puppet on the left or right. Proportion of time of looking toward the imitator or imitated puppet was calculated for each participant by dividing the time spent looking at the imitator or imitated divided by the sum of their total looking towards both puppets. Preceding the analyses, inter-coder reliability was checked by double-coding 10 different participants’ trials.

We reached an acceptable minimum of 95% reliability.

The primary dependent measures included (1) infants’ and toddlers’ first look direction (i.e., toward the puppet to the left or right) after the actor expressed distress, (2) the proportion of time that infants and toddlers spent looking at either puppet during the pause following the distress, and (3 and 4) the proportion of time infants and toddlers spend looking at the puppets during the ‘social preference test’ and the ‘non-social “general” preference test’.

We used JASP (JASP Team, 2024) to calculate Bayes Factors (BF) for the DVs described above. For Analysis 1 we pre-registered and performed a one-sided Bayesian binomial test comparing the likelihood of the data given the null hypothesis (that they look first at the imitator/imitated puppet less than half the time) to the alternative (that they looked first at the imitator/imitated puppet more than half the time). For Analyses 2–4, we performed a one sample Bayesian t-tests comparing the likelihood of the data given the null hypothesis (that they look at the imitator/imitated puppet less than half the time) to the alternative (that they look at the imitator/imitated puppet more than half the time). For the t-tests during the Anticipatory Looking trials, we used signed Wilcoxon t-tests after discovering that the distributions were not normal (see Supplementary materials). The Wilcoxon t-tests were not pre-registered, so we provide the student t-test results in the supplementary materials (the two analyses agree). The findings of more analyses, including tests for age effects and robustness checks, appear in supplementary materials.

We pre-registered a stopping rule because with Bayesian hypothesis testing a BF can be computed with each data point until a pre-determined threshold of evidential strength is reached (Wagenmakers et al., 2016). Following this design, the initial sample consisted of 18 infants and toddlers before a BF was computed. The study then stopped until either a BF_10_ of 10 was reached for the main experiment representing strong evidence for the alternative hypothesis or until 50 babies were tested (Kass & Raftery, 1995).

### Study 1 Results

#### Infants.

In the Actor Condition, infants looked first (22/32; BF_10_ = 3.96) and longer (M_actor_ = .633, SD_actor_ = .233, BF_10_ = 53.47) at the puppet who imitated the central character. In the target condition, infants looked first (26/33; BF_10_ = 118.2) and longer (M_target_ = .684, SD_target_ = .299, BF_10_ = 64.497) at the puppet who had been imitated by the central character. These anticipatory looks did not seem to be due to general interest, because infants did not look longer at the imitator or target puppet in any of the preferential-looking tests (Actor: M_speaking_ = .496, SD_speaking_ = .155, BF_01_ = 5.31; M_music_ = .525, SD_music_ = .154, BF_01_ = 3.52; Target: M_speaking_ = .456, SD_speaking_ = .218, BF_01_ = 2.61; M_music_ = .497, SD_music_ = .128, BF_01_ = 5.36; See Figure 1, C).

#### Toddlers.

In the actor condition, toddlers looked first (23/30; BF_10_ = 33.97) and longer (M_actor_ = .647, SD_actor_ = .279, BF_10_ > 1000) at the puppet who imitated the central character. Likewise, in the target condition, toddlers looked first (23/30: BF_10_ = 16.95) and longer (M_target_ = .688, SD_target_ = .292, BF_10_ > 1000) at the puppet who had been imitated by the central character. They did not look longer at the imitator or target in any preferential-looking test (Actor: M_speaking_ = .459, SD_speaking_ = .279, BF_01_ = 1.614; M_music_ = .486, SD_music_ = .105, BF_01_ = 4.01; Target: M_speaking_ = .536, SD_speaking_ = .128, BF_01_ = 1.94; M_music_ = .493, SD_music_ = .147, BF_01_ = 5.23).

### Study 1 Discussion

In Study 1, we found that infants and toddlers expected both social partners in an imitative interaction to respond to the other’s distress. This was true both when they had observed puppets who had imitated a central character and when they had observed puppets who had been imitated by the central character. These findings suggest that infants and toddlers interpreted the imitative interaction as evidence that the interactive partners were socially related to one another.

## STUDY 2

In Study 2, we tested a prediction that followed from the findings of Study 1: infants’ and toddlers’ expectations concerning the individuals involved in an imitative interaction should be limited to those parties. That is, if infants’ predictions about responses to distress are based on inferences of social relationships between social partners, they should not generalize to situations in which a new person who was not included in the initial interactions expresses distress. In contrast, if infants’ predictions were based on inferred social dispositions of the individuals in the imitative interaction, they should have no expectations about who would respond to the distress of the unfamiliar individual.

### Methods

See pre-registration: https://osf.io/e2dzw/?view_only=dca950dd78214fd4b215b8afc59af62b.

#### Participants.

30 infants (M_months_ = 11.90, SD = .24, range = 11.54–12.3, 20 female and 10 male) and 27 toddlers (M_months_ = 17.72, SD = .53, range = 16.64–18.51, 16 female and 11 male) participated in the study. One infant’s data was excluded from the target condition because of video quality; 1 infant’s data was excluded for the anticipatory trial in the target condition because they did not look at either puppet; 1 infant’s data was excluded from the actor anticipatory trial because they did not look at either puppet. One toddler’s data was excluded from the actor anticipatory trial because they did not look at either puppet.

#### Procedure and Materials.

The procedure was the same as in Study 1, except that in the Anticipatory test trial, the central character was replaced by a new human actor who was not featured in the imitative interactions during familiarization (See Figure 1, B).

#### Analysis and Stopping Rule.

For Study 2 we had pre-registered that we would stop after obtaining a Bayes Factor of 10. However, because we hypothesized that we would find evidence for the null, we stopped once a BF_01_ of at least 3 was reached (Schönbrodt et al., 2017). The asymmetric threshold was chosen as a conservative and commonly utilized option, with evidence for the null accumulating at a much slower pace (Wetzels et al., 2011; Schönbrodt & Wagenmakers, 2018, p. 133). The evidence was interpreted using classification tables, which provide a sensitive scale of interpreting Bayes Factors.

### Study 2 Results

#### Infants.

In the actor condition, infants did not look first (16/29; BF_01_ = 6.48) nor longer (M_actor_ = .465, SD_actor_ = .338, BF_01_ = 8.003) at the puppet who imitated the central character. Likewise, in the target condition, infants did not look first (10/28; BF_01_ = 10.42) nor longer (M_target_ = .481, SD_target_ = .360, BF_01_ = 6.05) at the puppet who had been imitated by the central character. Infants also did not look more at either puppet in any of the preferential-looking tests (Actor: M_speaking_ = .507, SD_speaking_ = .155, BF_01_ = 4.24; M_music_ = .499, SD_music_ = .154, BF_01_ = 5.96; Target: M_speaking_ = .456, SD_speaking_ = .218, BF_01_ = 4.93; M_music_ = .471, SD_music_ = .138, BF_01_ = 9.73; See Figure 1, C).

#### Toddlers.

In the actor condition, toddlers did not look first (14/26; BF_01_ = 3.89) nor longer (M_actor_ = .497, SD_actor_ = .261, BF_01_ = 5.27) at the puppet who imitated the central character. In the target condition, toddlers also did not look first (13/28; BF_01_ = 4.18) nor longer (M_target_ = .54, SD_target_ = .25, BF_01_ = 2.01) at the puppet who had been imitated by the central character. Toddlers also did not look longer at either puppet in any of the preferential-looking tests (M_speaking_ = .53, SD = .19, BF_01_ = 2.48; M_music_ = .52, SD = .13, BF_01_ = 2.36; M_speaking_ = .488, SD = .17, BF_01_ = 6.39; M_music_ = .474, SD = .117, BF_01_ = 10.112; See Figure 1, C).

### Study 2 Discussion

In Study 2, neither infants or toddlers had expectations about who would respond to the distress of a person who was not involved in the imitative interactions. This finding contrasts with the findings from Study 1, in which infants and toddlers’ exhibited expectations about the responses of the individuals involved in the imitative interactions. It provides further evidence that infants’ and toddlers’ inferences about imitators and their targets focus on the social relationship between those individuals in the interaction rather than on their individual dispositions.

## STUDY 3

In Study 3, we asked whether infants’ predictions about future interactions differed, depending on whether an action was likely to elicit a response from a stranger or from someone in an established relationship with the target. Specifically, we asked whether infants expected those involved in imitative interactions to respond to one another’s laughter. When a person expresses distress, their distress is most likely to elicit a response from others who know them. In contrast, when a person suddenly laughs, even strangers may respond to them (Devereux & Ginsburg, 2001; Owren & Bachorowski, 2003). In Study 3 we tested this prediction only in infants, because we found positive evidence that age did not affect the results in Studies 1 and 2 (see Supplementary Materials). We also sought to replicate our findings from Study 1. We randomly assigned infants to one test condition, which was identical to the test conditions in Study 1, and one control condition, in which the central character laughed instead of expressing distress during the Anticipatory looking test trials.

Study 3 was pre-registered here: https://osf.io/e2dzw/?view_only=dca950dd78214fd4b215b8afc59af62b.

### Methods

#### Participants.

66 infants participated in the study (M_months_ = 11.92, SD = .24, range = 11.47–12.5 months, 32 female, 22 male, 11 parents did not specify). See pre-registration for the stopping rule. 20 infants’ target conditions were not included in the analysis because of bad video quality (N = 13); because the baby didn’t look at either puppet (N = 6) and experimenter error (the experimenter neglected to ask the parent to turn off their video (N = 1). Eleven infants’ actor conditions were not included in the analysis because of bad video quality (N = 8), because the infant did not look at either puppet in the actor condition (N = 2) and because of experimenter error (the experimenter forgot to ask the parent to turn off their video (N = 1).

#### Procedure and Materials.

The procedure was the same as in Study 1, except that in the Anticipatory test trial, the central character expressed laughter before putting their head in their hands and down on the table. We also conducted but did not code the two preferential-looking tests, since in Studies 1 and 2 we found null results across two studies, two conditions, and two age groups.

### Study 3 Results

In line with our hypothesis, infants did not expect the imitative partners to respond to the central person’s laughter. In the actor condition, infants did not look first (8/24; BF_01_ = 10.17) nor longer (M_actor_ = 0.332, SD_actor_ = .412, BF_01_ = 11.89) at the imitator after the central character laughed. Likewise, in the target condition, infants did not look first (4/21; BF_01_ = 14.5) nor longer (M_target_ = .351, SD_target_ = .403, BF_01_ = 10.511) at the puppet who had been imitated by the central character after the central character laughed (See Figure 1, C).

Replicating Study 1, in the actor condition during the distress test trial infants again looked first (24/30; BF_10_ = 116) and longer (M_actor_ = .754, SD_actor_ = .299, BF_10_ = 169.246) at the puppet who had previously imitated the central character. Likewise, in the target condition, in the distress test trial, infants looked first (19/25, BF_10_ = 14.59) and longer (M_target_ = 0.804, SD_target_ = 0.289, BF_10_ = 501) at the puppet who had been imitated by the central character.

When comparing across these conditions, we found strong evidence in both the target and actor conditions that infants looked first more often (Bayesian Contingency Table, Actor: BF_10_ = 129.51; Target: BF_10_ = 633.83) and longer (Bayesian Independent Sample T-test; BF_10_ = 329.87; Target: BF_10_ = 401.70) at the puppet that was involved in the imitative condition after central character expressed distress compared to when the central character laughed.

## GENERAL DISCUSSION

In three studies, we find evidence that 12-month-old infants infer social relationships from observed acts of imitation. In Studies 1 and 3, participants looked first and longer at imitators compared to non-imitators after the imitator’s social partner expressed distress. Likewise, participants looked first and longer at puppets who were imitated, compared to puppets who were not imitated, after the imitated puppet’s social partner expressed distress. In Study 2, we reasoned that if participants’ anticipatory looks were based on inferred relationships, they should have no expectations when a person uninvolved in the initial interactions expressed distress. However, if their anticipatory looks were based on inferred dispositions, they should expect the puppets to respond to a person uninvolved in the initial interactions. We found evidence for the former: when a new person expressed distress, infants and toddlers looked neither first nor longer at the puppet who was involved in the imitative interaction. Finally, in Study 3, while replicating the results in Study 1, we found evidence that infants do not expect responses to laughter: a social behavior that often elicits responses from strangers. After infants observed the same imitative interactions, they did not expect imitators nor their targets to respond to the laughter of their social partner. Taken together, these results suggest that by 12 months of age, infants infer relationships between the individuals involved in imitative interactions.

Why might infants use imitation to make inferences about social relationships? As discussed in previous work, imitation is a good cue to social attention, because the imitator both recognizes their social partner’s goal and adopts it (Powell, 2022). One may ask, however, whether infants were given evidence that the individuals who vocally imitate each other speak the same language, whereas individuals who don’t imitate one another do not speak the same language. If they do, could this have driven their inferences^1^? Infants prefer to look at individuals who speak the same language as their caregiver (Kinzler et al., 2007). Infants and children expect people who speak the same language to be friendly toward one another (Liberman et al., 2021), and they themselves prefer individuals whose speech is pronounced in the accent of their native language, whether or not the speech occurs in that language (Kinzler, 2021; Kinzler et al., 2007, 2009; Shutts et al., 2009, 2010). In our study we give infants evidence that the individuals speak the same language by showing that they say the same word (a word that is likely unknown to the baby). One reason to think that babies might *not* interpret this as evidence that the two individuals speak the same language is that in other studies they prefer an imitator to a non-imitator even when the babies themselves are not familiar with the computer-generated sound or spoken word (Powell & Spelke, 2018b; Thomas et al., 2020). Infants use prosody to distinguish their native language from other languages from birth, but that the process of learning the sounds and meanings of words develops slowly over the first 14 months, even for the most common words that infants hear (e.g., Bergelson, 2020; Bergelson & Aslin, 2017). Thus, it seems unlikely that a baby would prefer someone who spoke the same language as someone else without any connection to the baby herself. In line with other arguments that imitation is a way of signaling adopted utility and following Relational Models Theory, we propose that the reason why infants use imitation as a cue of relationships is that it is a way that someone can make themselves more ‘as one’ with someone else, signaling attention or an acknowledging shared fate. Thus, imitation relates to other cues such as having the same clothing, sharing saliva, or interpersonal synchrony, that have also been implicated in infants social preferences and expectations of further interaction (Cirelli et al., 2014; Jin & Baillargeon, 2017; Thomas, Woo, et al., 2022; Ting et al., 2019).

Moreover, whilst infants can discriminate between two different languages, including those that are unfamiliar to them, their early discrimination between languages depends on whether the languages differ in prosodic structure (Bertoncini et al., 1995; Kinzler, 2021). As newborns, for example, infants can discriminate French from Japanese, but not French from Italian (Nazzi et al., 1998). With age, infants become increasingly able to differentiate a familiar language from another, but they still struggle with similar languages that are unknown to them (Nazzi et al., 2000). In our study, we used unknown words that were similar or identical in number of syllables as well as identical repetition (I.e., Kaza, Kaza and Piku, Piku). Such rhythmic similarity makes it unlikely that infants viewed the sounds as words in two different languages.

The present findings differ from those found in previous studies of younger infants, suggesting that infants’ inferences may change throughout the first year. In the current studies, both 12-month-old infants and 16- to 18-month-old toddlers displayed the same behavior. However, four-month-old infants, who were presented with imitative interactions performed by animated characters, developed asymmetrical expectations concerning the characters’ future social behavior (Powell & Spelke, 2018a). When reasoning about imitators, they expected the imitators to approach their social partners (i.e., the targets of their imitation). However, they did not expect the targets of imitation to approach their social partners (i.e., the individuals who had imitated them). While comparisons across studies that use different displays and different dependent variables should be interpreted with caution, it is possible that the difference in the two findings reflects a developmental change from 4 to 12 months: younger infants may have more limited expectations about the targets of social actions. If this is true, it would perhaps be unsurprising given that the most relevant relationships to a 4-month-old infant are caregiving relationships, which involve many asymmetrical obligations and interactions. It is possible that over the first year of life, as infants become more competent social partners, they come to have more symmetrical expectations about the initiators and targets of imitative interactions. Therefore, our results extend prior research by providing evidence for developmental changes, between 4 to 12 months of age, in expectations for targets of imitation.

The present findings differ from the implications of previous research in a further way: In our studies, infants’ inferences were constrained to the people involved in the interaction. In previous studies with infants of the same age, in contrast, infants reach for characters who have imitated other characters. Why would they do this, if imitation were not viewed, in part, as indicative of a prosocial disposition? First, it is possible that infants expect imitators to have some social dispositions—they may be more friendly, helpful, or engaging with others—but not other social dispositions—they may not be more responsive to the distress of unknown others. However, the findings of Study 3 cast doubt on the hypothesis that infants expect imitators to be more friendly, because infants did not expect imitators to respond more readily to laughter even of their social partners.

Second, infants who direct social actions at those who have imitated others may see themselves as part of the initial interactions. In almost all studies that investigate social evaluations, the characters involved in the interactions direct attention toward the infant participant. A key difference therefore between the infant in these experiments and the adult who was not part of the initial interactions, but expressed distress in Study 2, is that the uninvolved individual was not shown observing the initial interactions. Future studies could test whether infants expect imitators to respond to the distress of someone who had observed, but not been involved in, the initial interactions.

Finally, infants’ tendency to look at and reach for imitators may be motivated by their interest in those individuals and their potential future behavior rather than by inferences about the potential social value of those individuals to the infant. For example, infants may view imitators as more competent rather than more prosocial, because their behavior requires a capacity to plan actions with second order goals (Spelke, 2022). This would not necessarily lead to predictions about who would be more likely to respond to distress. Future studies could also investigate whether infants expect helpers and imitators to be more competent.

Why might infants at this age be motivated to map the relationships of people around them? First, as much as it is useful to predict the behavior of people, much of people’s behavior, especially their social behavior, is dictated by their social relationships (Fiske, 1992; Thomsen & Carey, 2013). Moreover, like all other primates, the network of human relationships that surround infants has implications for their wellbeing and survival (Hrdy, 2009; Hrdy & Burkart, 2022; Silk, 2007). For human infants, the ability to map social relationships might be especially important as infants depend on many caregivers beyond their mothers (Hrdy, 2009; Hrdy & Burkart, 2022), and children depend on many people to learn the vast amount of culturally specific information that they need in order to be competent members of their social groups. Understanding the relationships between others can help infants and children make decisions that would help them interact with potential caregivers or teachers (Hrdy, 2009; Thomas, Saxe, et al., 2022; Thomas, Woo, et al., 2022). Thus, the fact that infants use a common cue of relationships to make inferences about relationships suggests that humans are equipped to map the social networks connecting the people around them from a young age.

What implications do these results have for current theories on socio-emotional development? First, they support claims that humans are endowed with innate abilities to make inferences about the social relationships connecting the individuals in the child’s environment (Fiske, 1992; Hirschfeld, 1999; Kaufmann & Clément, 2014; Thomas, n.d.; Thomsen & Carey, 2013). This claim is in contrast to claims that humans are endowed with an innate moral core, which have focused on infants’ ability to evaluate others in terms of their individual disposition to prosociality. The infants in the present experiments did not expect that imitators would behave prosocially to a new individual, nor did they expect them to behave prosocially in all situations. Rather, their inferences and expectations seemed to be based on inferences about relationships. People’s ideas about morality may depend on these inferences (Rai & Fiske, 2011). Thus, it is possible that infants *do* have an ‘innate moral core’ but that it is intertwined with the relationships they infer. Moreover, it is possible that infants do infer prosocial traits from observing many actions, but that imitation is not one of them. More work can be done to investigate when infants make inferences about traits versus relationships as well as the role of social groups as opposed to interpersonal relationships in infants’ understanding of social interactions.

In summary, three experiments provide evidence that both 12-month-old infants and 16- to 18-month-old toddlers view imitation as a social behavior that is indicative of social relationships rather than individual dispositions. Infants’ expectations of responses to distress were constrained to the individuals involved in the initial interactions. Moreover, infants did not expect those involved in the imitative interactions to respond to social behaviors that frequently occur between strangers, as they did not expect the imitators or individuals who were imitated to respond to the laughter of their social partners. Finally, infants’ representations of the relationships connecting individuals were abstract: they connected imitation to a very different type of social behavior (responses to distress) and applied their knowledge of imitation to learn about new social relationships.

## ACKNOWLEDGMENTS

Thank you to Beyza Kazan, Hayley Rhorer, María Garcia Garcia, Gabriel Chisolm, and Sam Gregory for help with data collection for help with data collection and creation of the events presented to infants. Thank you to the members of the Cambridge Writing Workshop who gave feedback on many versions of this manuscript. This research was funded by NIH National Research Service Award 1F32HD096829 and the Center for Brains, Minds, and Machines, funded by the National Science Foundation STC Award CCF-1231216 and Siegel Foundation Award S4881. AJT was supported by the Templeton Foundation, TWCF-2021-20639 Understanding Caregiving: Biology, Psychology, & Policy during the writing of this manuscript. Parts of this manuscript were written for VK’s senior thesis at Bath University.

## AUTHOR CONTRIBUTIONS

VK: Conceptualization, Methodology, Data collection, Data management, Writing Reviewing & Editing, Writing Original Draft, Analysis; ES: Conceptualization, Funding, Writing Reviewing & Editing, Methodology; AT: Conceptualization, Methodology, Data collection, Data management, Writing Reviewing & Editing, Writing Original Draft, Data Visualization, Analysis.

## DATA AVAILABILITY STATEMENT

Data is available: https://osf.io/e2dzw/.

## Note

^1^ We thank an anonymous reviewer for suggesting this interpretation.

## Supplementary Material


