# Supplementary material for: Infants Infer Social Relationships Between Individuals Who Engage in Imitative Social Interactions
Source: Open Mind (Camb). 2024 Mar 5;8:202–16. doi: 10.1162/opmi_a_00124 (PMC10932586; doi:10.1162/opmi_a_00124)
Supplement: Supplementary file 1 [file opmi-08-202-s001.docx]

Supplementary Materials

**Coding, Stopping Rule and Analyses**

We conducted Bayesian analyses in the program JASP (JASP Team, 2020). As such, the Bayes Factors (BF) reported throughout the manuscript evaluate the conditional probability between two competing hypotheses (i.e., the null and the alternative), quantifying the degree to which the data demand a change in prior beliefs concerning the hypotheses' relative plausibility; Jeffreys, 1961). Accordingly, as BFs do not force an all or one decision and are updated as data is given, they allow for preferential stopping (Wagenmakers et al. 2016). Following this flexible stopping rule, at least 18 infants and toddlers and no more than 30 were tested, or until a BF_10_ of 10 was reached for the main experiment, representing strong evidence for the alternative hypothesis (i.e., the data are 10 times more likely under the alternative than the null; Kass & Raftery, 1999; Mani et al. 2020). For Study 2 we use the same stopping rule, except, because we hypothesized the null, we stopped once a BF_01_ of at least 3 was reached, (Schonbrodt et al. 2017). The asymmetric threshold was chosen as a conservative and commonly utilized option, with evidence for the null accumulating at a much slower pace (Wetzels et al., 2011; Schönbrodt & Wagenmakers, 2018, p. 133). The evidence was interpreted against Doorn et al’s (2019) classification tables, which provide a sensitive scale of interpreting BF’s.

To test whether the first looks differed from chance, we used a Bayesian binomial test. In the first experiment, we pre-registered one-sided tests given the directional hypothesis and in the second experiment, it was two-sided, because the null was predicted. As such, the null hypothesis for these tests was that infants and toddlers would look equally often toward the imitator/imitated as they would toward the non-imitator puppets. The alternative for experiment 1 was that infants would look first more often toward the imitator/imitated puppet. In experiment 2, the alternative was that infants would look more or less often toward the imitator/imitated. Moreover, to test whether infants’ spent a larger proportion of time looking at one of the puppets, we used a Bayesian one-sample t-test. Accordingly, the test was one-sided for the Anticipatory Looking Trial because we predicted that infants would look first at the imitator and imitated puppet, and two-sided for the Social Preference Trial and Silent Preference Trial, as no predictions were made ahead of time about which puppet babies would look longer at. The tests were two-sided for all trials in Study 2, and the laughter condition for Study 3 as the null was predicted.

Prior to these statistical analyses, we assessed looking time proportions for normality using a Shapiro-Wilk test in JASP, and found that the data was not normal. Based on this, the Bayesian student t-test was replaced by a Wilcoxon signed-rank test which does not require data to be normally distributed and compares the sample median to the hypothetical median as opposed to the means (Benavoli et al., 2014). Moreover, a default uniform Beta (1,1) prior distribution was selected for all binomial tests and a default prior Cauchy distribution with spread r set to 1/√2 was selected for the t-tests. Defaults were chosen as a conservative approach to data analysis (Mani et al. 2020). For the same reason, a sensitivity analysis was conducted, to test the consistency of the BFs over a range of prior specifications and thus, their robustness. The results of this robustness test are reported below for each experiment.

# Study 1 additional analyses

## Study 1 Assumption of Normality Test

Using JASP, we performed a Shapiro-Wilk Test for normality and found a significant deviation in both age groups. For 12-month-olds (Shapiro-Wilk Test : *Wtarget*=.878, *p*=.001; *Wactor*=.926, *p*=.03). For 18-month-olds (Shapiro-Wilk Test: *Wtarget*=.931, *p*<.001; *Wactor*=.919, *p*=.003).

## Study 1 pre-registered analyses

The pre-registered analyses, using a student t-test agreed with the Wilcoxin tests reported in the manuscript. (For infants, Target: BF_10_=25.62; Actor: BF_10_=38.50. For Toddlers, Target: BF_10_=25.62; Actor: BF_10_=38.50)

**Study 1 Age Effects**

Using an independent sample t-test, we found positive evidence that infants and toddlers performed similarly in both conditions in the anticipatory looking trials (Actor: BF_01_ =3.87 ; Target: BF_01_ =3.79).

## Study 1 robustness tests

The following robustness tests were performed for the Anticipatory Looking Test Trials.

### Infants

| **Actor Condition** | |
| --- | --- |
| **Bayes Factor Robustness Check**  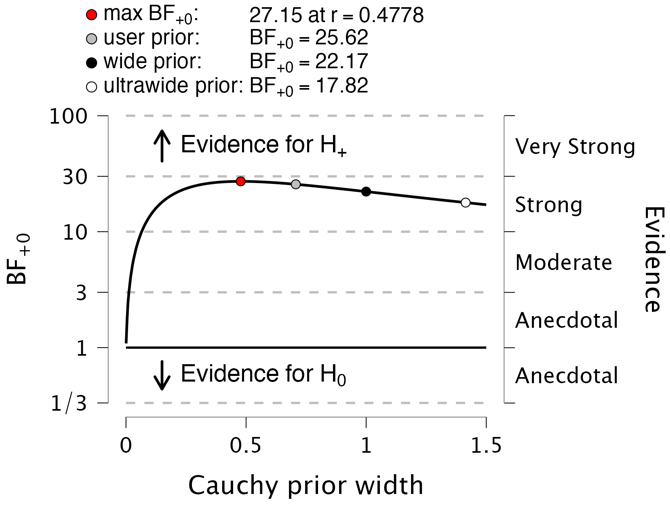 | **Sequential Analysis**  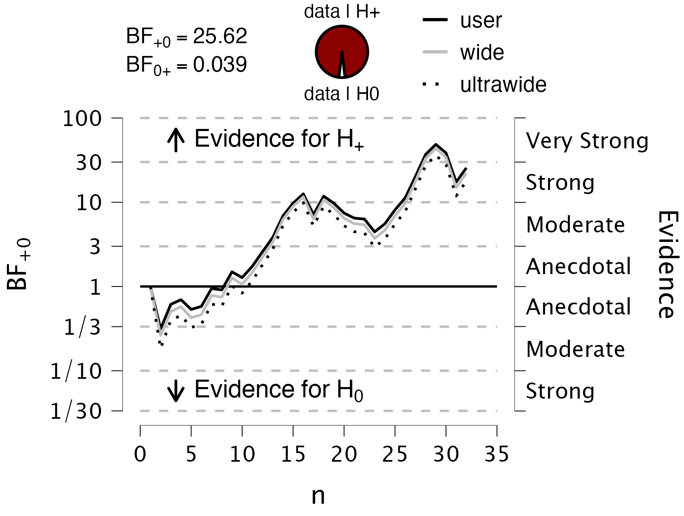 |
| **Target Condition** | |
| **Bayes Factor Robustness Check**  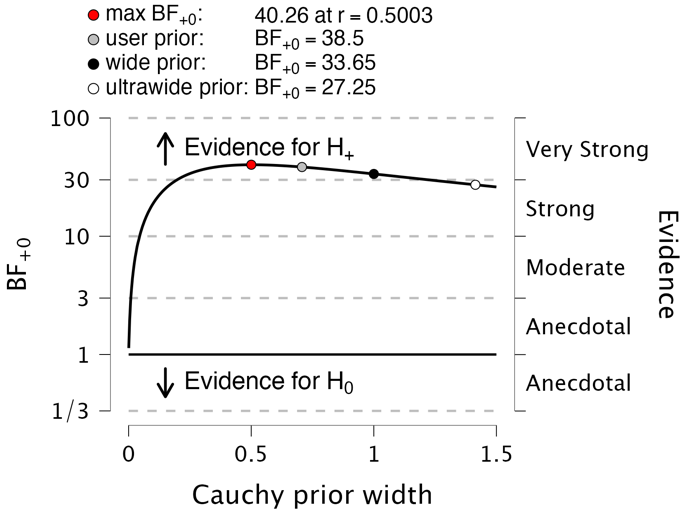 | **Sequential Analysis**  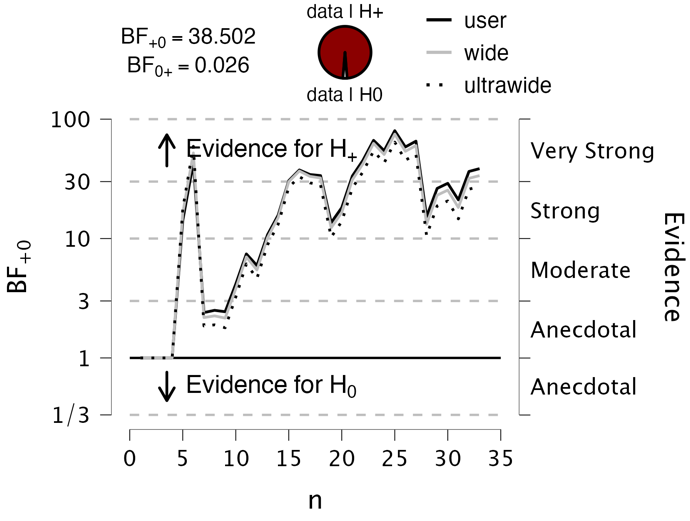 |

*Toddlers*

| **Actor Condition** | |
| --- | --- |
| **Bayes Factor Robustness Check**  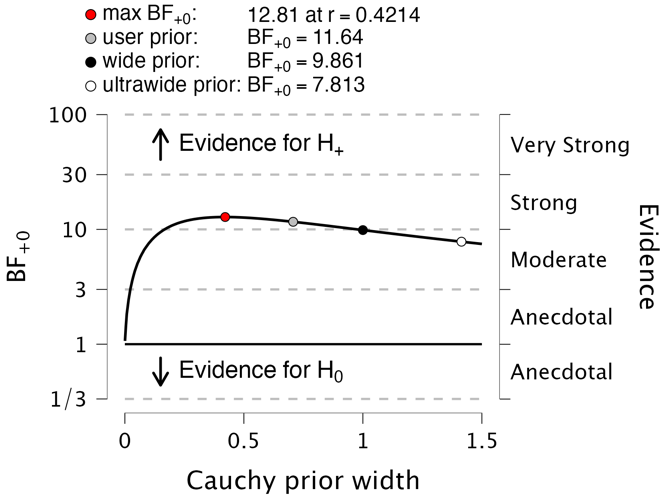 | **Sequential Analysis**  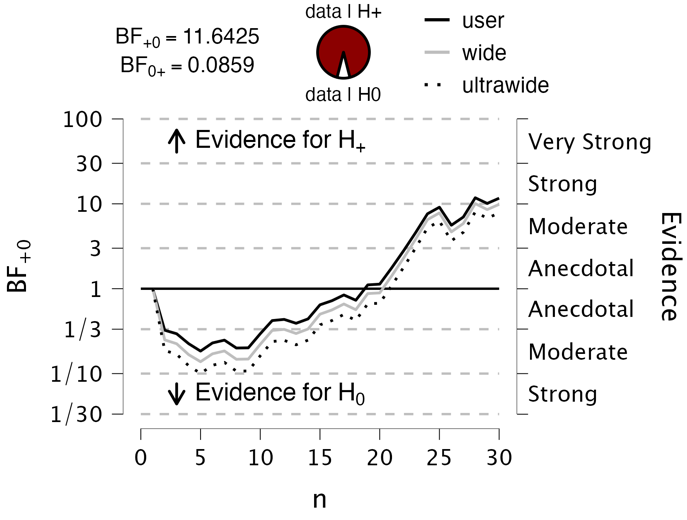 |
| **Target Condition** | |
| **Bayes Factor Robustness Check**  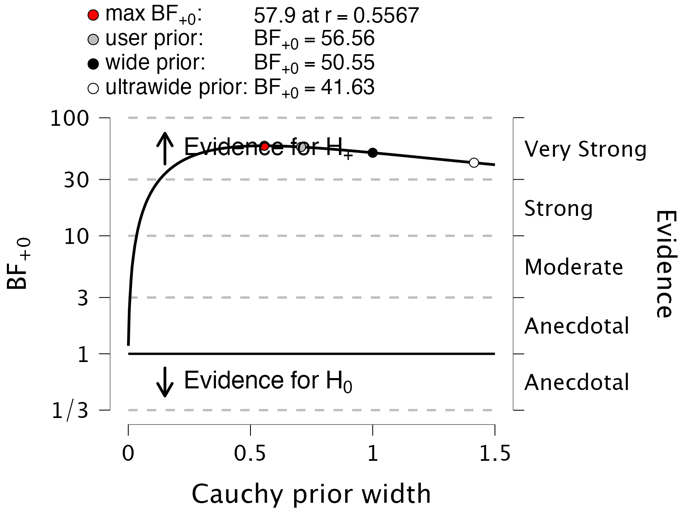 | **Sequential Analysis**  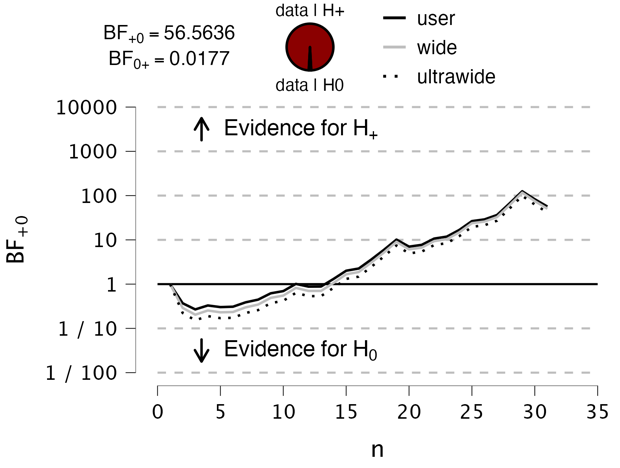 |

# Study 2 Additional Analyses

## Study 2 Assumption of Normality Test

Using JASP, we performed a Shapiro-Wilk Test for normality and found a significant deviation in both age groups. For 12-month-olds (Shapiro-Wilk Test: Target: *W*=.902, *p*=.011; Actor: *W*=.926, *p*=.043). For 18-month-olds (Shapiro-Wilk Test: Target, *W*=.931, *p*<.001; Actor, *W*=.919, *p*=.003).

## Study 2 pre-registered analyses

Our pre-registered analyses, using a student t-test, agreed with the Wilcoxon tests reported in the manuscript (Infants: Target: BF_01_=6.095; Actor: BF_01_=7.37; Toddlers: Target: BF_01_=3.142; Actor: BF_01_=4.819)

**Study 2 Age Effects**

As in Study 1 we find positive evidence that infants and toddlers performed similarly (Actor: BF_01_ =3.44 ; Target: BF_01_ =3.57).

**Study 2 Robustness Checks**

*Infants*

| **Actor Condition** | |
| --- | --- |
| **Bayes Factor Robustness Check**  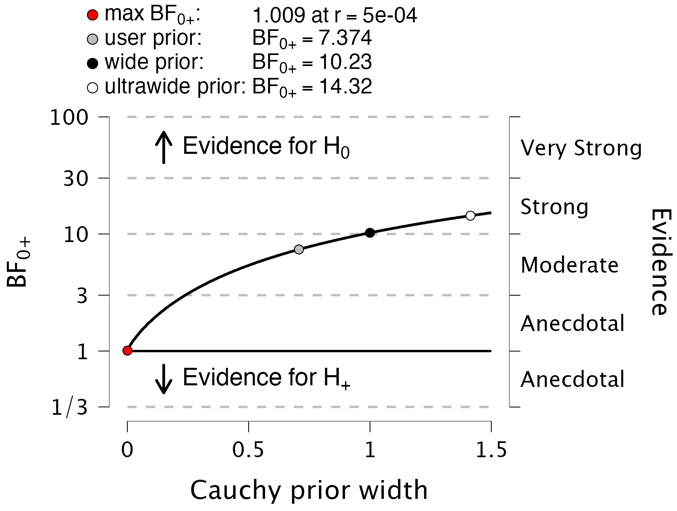 | **Sequential Analysis**  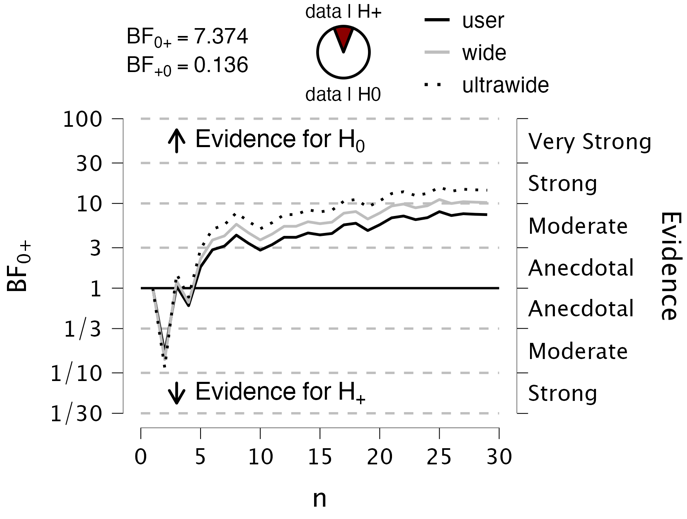 |
| **Target Condition** | |
| **Bayes Factor Robustness Check**  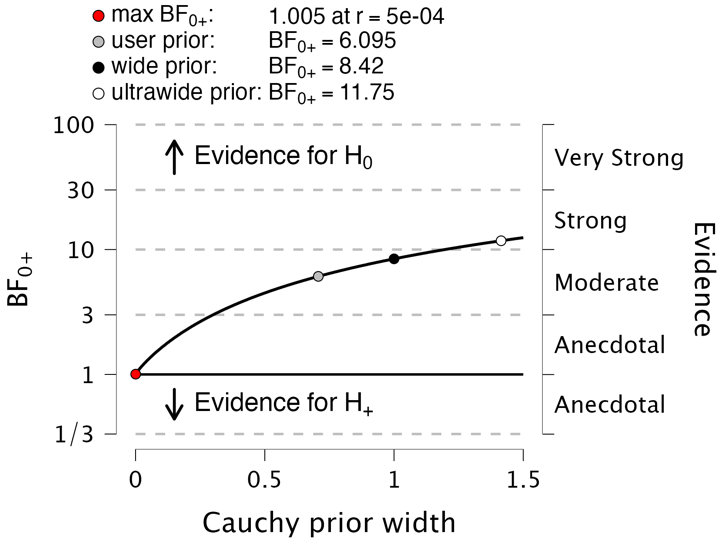 | **Sequential Analysis**  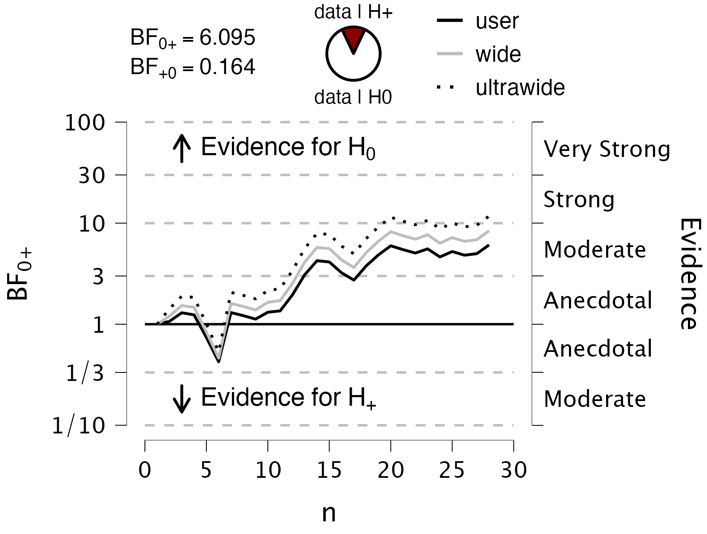 |

*Toddlers*

| **Actor Condition** | |
| --- | --- |
| **Bayes Factor Robustness Check**  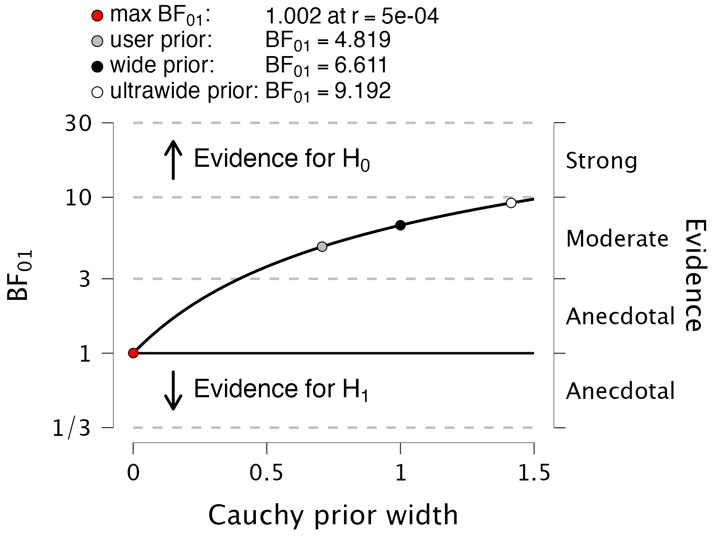 | **Sequential Analysis**  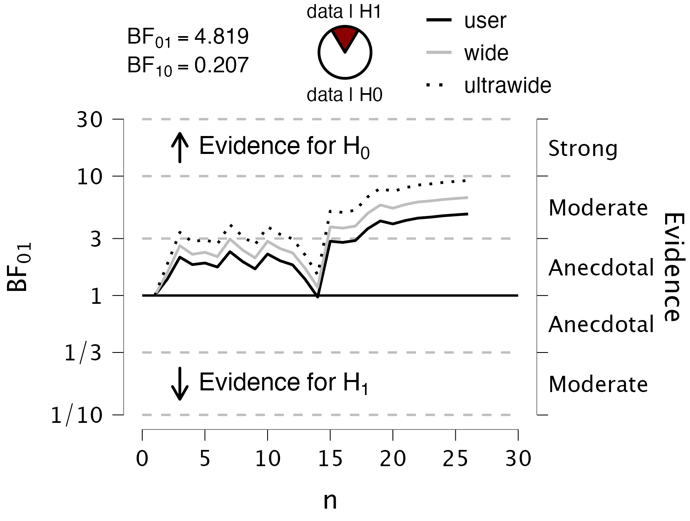 |
| **Target Condition** | |
| **Bayes Factor Robustness Check**  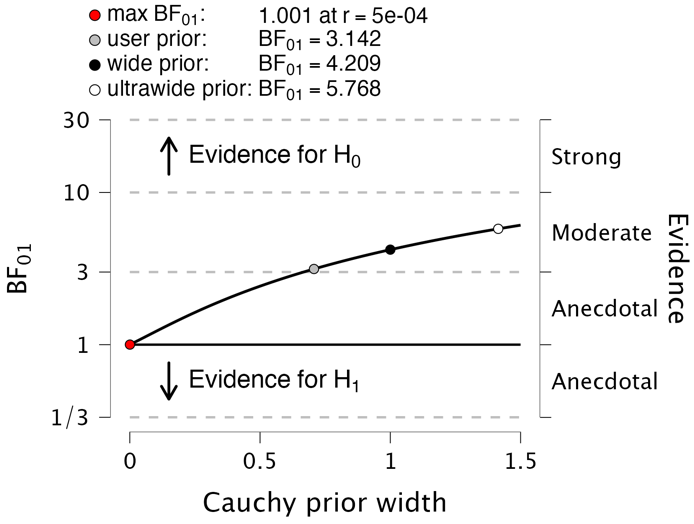 | **Sequential Analysis**  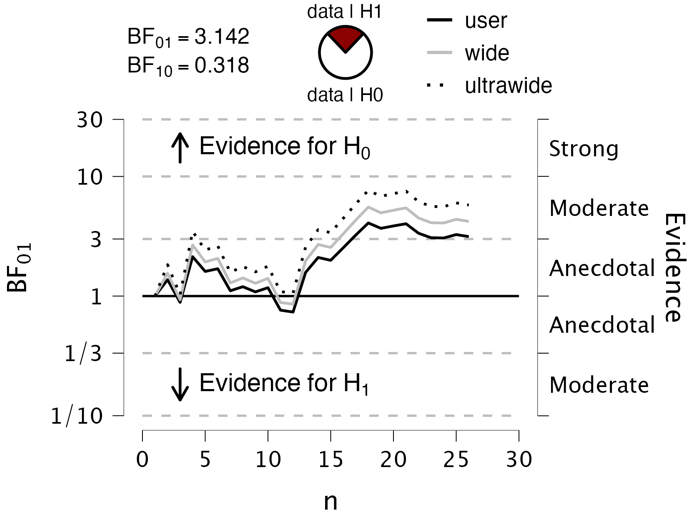 |

# Study 3 Additional Analyses

## Study 3 Assumption of Normality Test

Using JASP, we performed a Shapiro-Wilk Test for normality and found a significant deviation in both the laughter test trials and the distress test trials. For laughter test trials (Shapiro-Wilk Test: Target: *W*=.902, *p*=.011; Actor: *W*=.744, *p*<.001). For the distress test trials (Shapiro-Wilk Test: Target, *W*=.729, *p*<.001; Actor, *W*=.919, *p*=.003).

## Study 3 pre-registered analyses

Our pre-registered analyses, using a student t-test, agreed with the Wilcoxon tests reported in the manuscript (Distress: Target: BF_10_=3097; Actor: BF_10_=740.72; Laughter: Target: BF_01_=10.511; Actor: BF_01_=12.39)

**Study 3 robustness checks**

**Laughter Test Trial**

| **Actor Condition** | |
| --- | --- |
| **Bayes Factor Robustness Check**  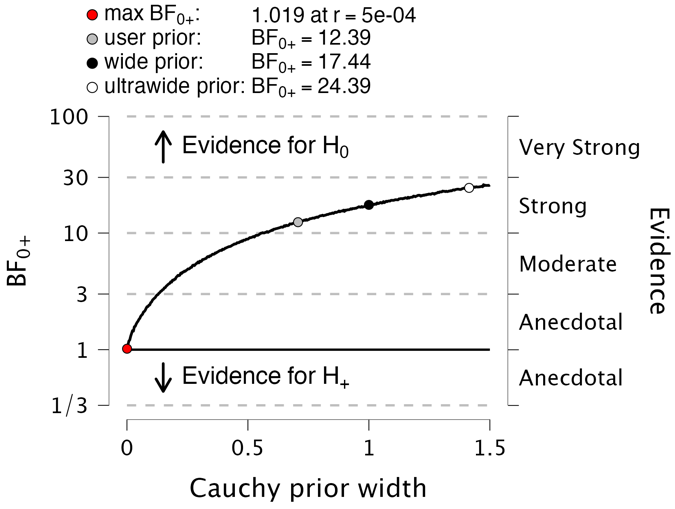 | **Sequential Analysis**  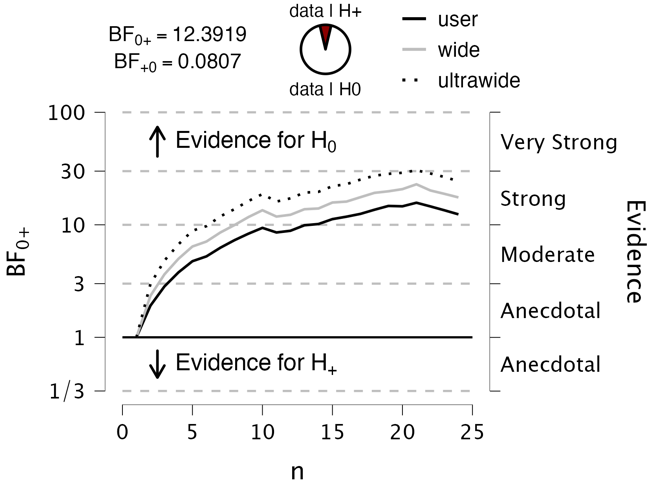 |
| **Target Condition** | |
| **Bayes Factor Robustness Check**  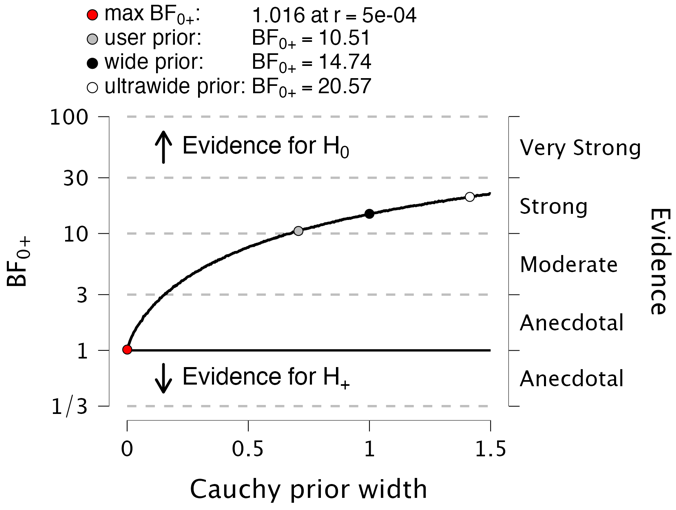 | **Sequential Analysis**  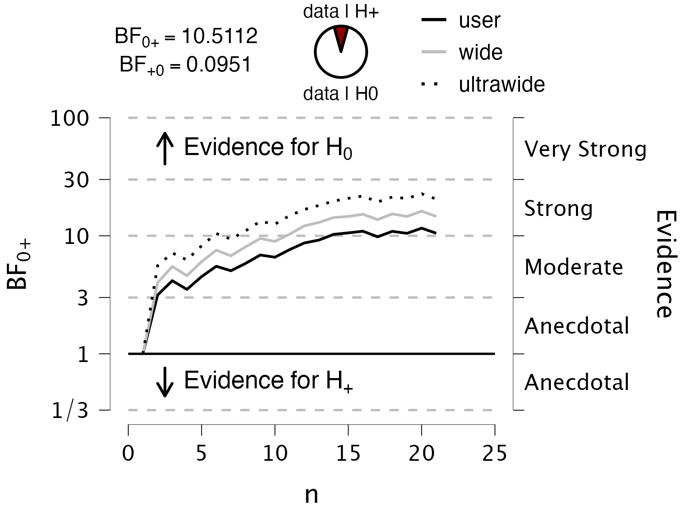 |

**Distress Test Trial**

| **Actor Condition** | |
| --- | --- |
| **Bayes Factor Robustness Check**  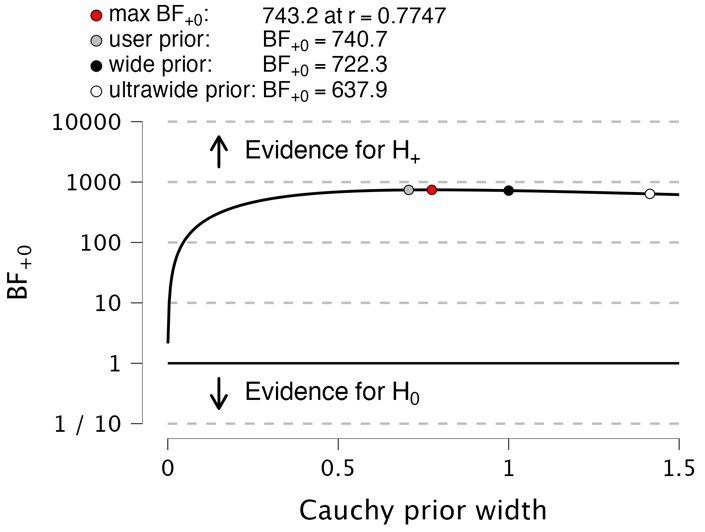 | **Sequential Analysis**  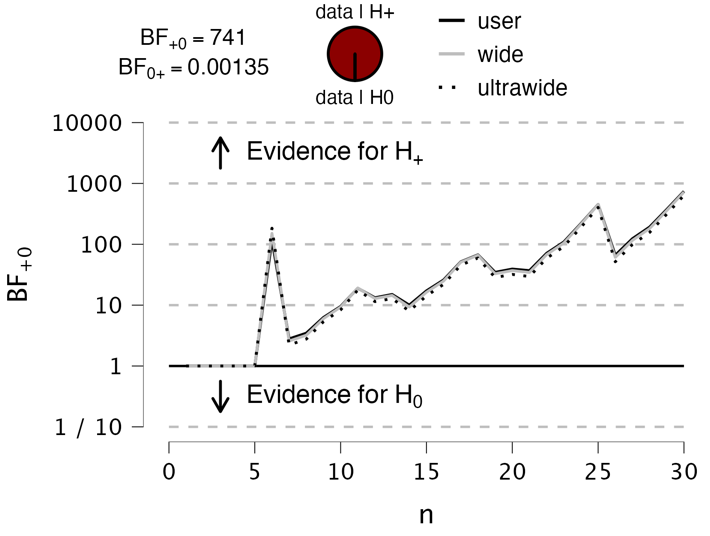 |
| **Target Condition** | |
| **Bayes Factor Robustness Check**  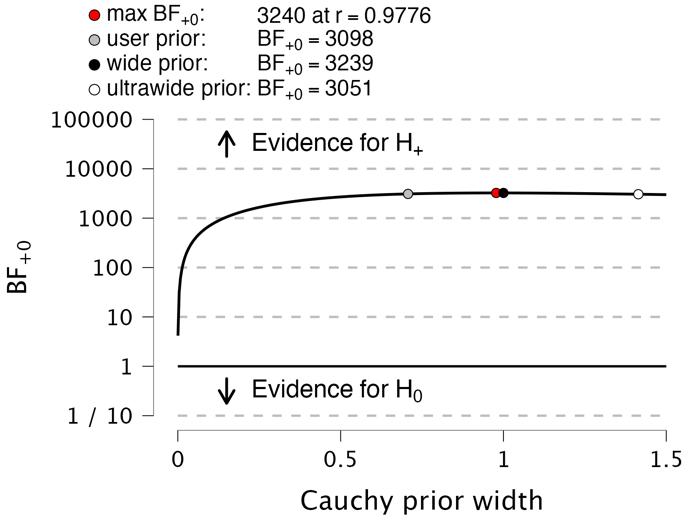 | **Sequential Analysis**  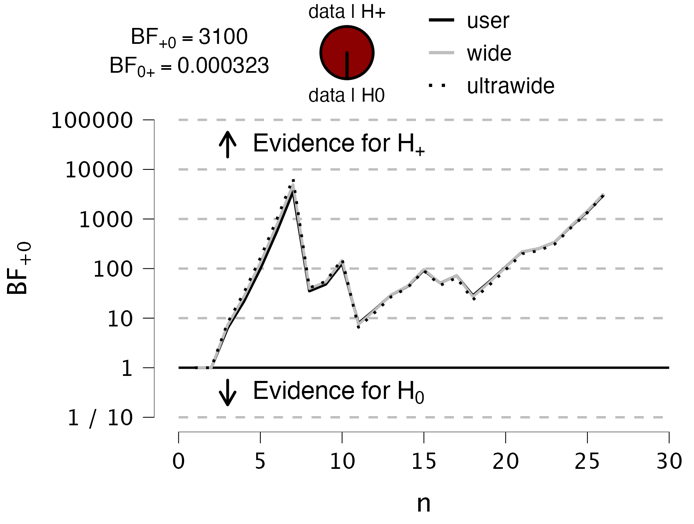 |
